# Pedagogy in the time of COVID-19

**DOI:** 10.3325/cmj.2020.61.211

**Published:** 2020-06

**Authors:** Carol P. Poe

**Affiliations:** University of Georgia, College of Public Health, Athens, GA, USA *cpcotton@uga.edu*

The shock that came from the impact of COVID-19 on education and teaching has been like no other. Even though we see ourselves in higher education as well versed in the use of technology, and are certainly encouraged to utilize all technological advances put at our disposal, which are many, the imperative to immediately take all in-class instruction online was quite a disturbance. This has been felt dramatically in public health and medical education, where technology cannot, and certainly must not, replace social interaction. This pandemic challenges us as professional educators like no other event during our lifetime.

Online instruction is not a hobby. Done well, it is a finely honed skill, practiced over many semesters, just as we would with classroom strategies and skills. To take a class online with no training is not fair to either the faculty or the students. There are only a handful of faculty members who are truly experts in online instruction at this point, and after a rough couple of weeks, these people were made available to struggling faculty for assistance. As the semester progressed, more and more resources were made available to faculty to bring their skills up to a comfortable level for both students and teachers. However, the prospect of continually teaching online or partially online is daunting and for me, mostly dissatisfying.

Another problem that was encountered focused on where exactly each class was in the progression of education when the in-class instruction stopped and changed to distance learning. A class that is focused on PowerPoint slides and lecture is much easier to transfer to the online realm than is a class predicated on participation, group work, presentations, and hands-on experiences. Student presentations were particularly problematic because the students had prepared their work for in-class delivery, using the resources available in the classroom. A new deadline had to be determined for this work to ensure that students were given a fair opportunity to transform their work, keeping the quality level high, for presentation online. Some students were very comfortable with this and other students were not. The question was how to give the students who needed help with this transition time to do so, without penalizing the students who were all ready to move online. Essentially, every class became divided by technological ability.

The main obstacles to overcome were 1) adequate training for faculty so that pedagogical integrity would be maintained, 2) reestablishing reasonable expectations for student work, 3) equitable evaluation of all student work, and 4) maintaining student-faculty relationships.

Training for faculty was ramped up within a reasonable amount of time, and all resources at the University’s disposal were made available for faculty to access. The quality of online teaching runs the gamut just as face to face instruction does. Some faculty members are just better teachers than others, and no amount of technology or support is going to change that.

Reestablishing reasonable expectations for student work was a bit more problematic, in that it had to be decided quickly whether to move or maintain deadlines, and students complained that the work load seemed greater online when the deadlines were not modified. Notwithstanding student complaints, we had to understand that the students too were going through a seismic change in not only their education, but their living situations, their work, their finances, and their family concerns. At this time, students were not solely focused on their academic work, and some students’ life situations were exponentially more complicated than others. Therefore, some students just could not do all this under the time pressure of course deadlines.

Equitably evaluating student work is difficult when students are asked to change their work products, essentially changing how they themselves approach their work, on a moment’s notice. Some students adapt well and others do not. We must be careful to not grade the student’s adaptability, rather than their actual work product. In this area, resources were also made available for students whose online skills were lacking. Some projects or hands-on experiences had to be scrapped altogether because they simply were not suitable for online submission or were too cumbersome to move online, or simply could not be equitably substituted with anything online. Seeing a photograph of something is not the same as holding a tactile piece of work in your hand, and grading it fairly is much more difficult.

Lastly, maintaining student-faculty relationships was the most important part to me. Students often approach the podium with questions before or after class; students who are too shy to speak up in class. Hearing a student’s tone of voice, seeing the confusion or delight on their face, having a quick check on work, is incredibly helpful and important in the educational process and in building relationships. This mostly was lost online, or at least was very time consuming. Students queue after class and each student can be supported quickly. Online, even face-to-face online, takes much more time and the distance can feel like real distance in a relationship. I found myself spending more time with a student on the phone than I would have in the classroom, asking questions about their health or their family, trying to reestablish the relationship that was being threatened by distance.

The benefits of this cataclysmic shift are that we are all learning new skills; new educational skills, and new people skills. We are realizing that faculty and students are not just the people we see in class, but they have lives outside of class that affect their work. We must be cognizant of these outside factors to fully understand our colleagues and our students. When students and faculty come back into class, higher education will have changed. If we approach each other with these newly learned realizations, it could enrich the learning environment.

